# Statins: Masked anti-epileptic warriors

**DOI:** 10.1016/j.amsu.2022.104277

**Published:** 2022-07-31

**Authors:** Govinda Khatri, Priya, Ayush Kumar, Mohammad Mehedi Hasan

**Affiliations:** a Dow University of Health Sciences, Karachi, Pakistan; b Aga Khan University Hospital, Karachi, Pakistan; c Department of Biochemistry and Molecular Biology, Faculty of Life Science, Mawlana Bhashani Science and Technology University, Tangail, Bangladesh

Statins, such as Atorvastatin and Fluvastatin, are the most frequently prescribed medications for hypercholesterolemia. They are responsible to inhibit the key enzyme of cholesterol biosynthesis i.e., 3-hydroxy-3-methyl-glutaryl-coenzyme A reductase (HMG-CoA). HMG-CoA reductase reduces HMG-CoA to Mevalonate which is an important precursor molecule of cholesterol. Additionally, statins have a minor role as anti-inflammatory and anti-oxidants [1]. It is because of these properties that the CDC recommends the use of statin drugs for patients with a high risk of heart attack/stroke, previous history of heart attack/stroke, high LDL cholesterol, and occasionally diabetics [2]. However, just like most of the routinely prescribed medications, these too come with few adverse effects. Myopathy is the most common side effect of statins and in a few cases, it may result in rhabdomyolysis. Hepatotoxicity, peripheral neuropathy, and impaired myocardial contractility are a few other uncommon adverse effects that are associated with the use of statins [3]. Recently, an interesting finding has been observed in a few clinical trials where statins have been recognized for their anti-epileptic properties and this may be a major breakthrough in the treatment of brain insults, that too by a widely prescribe cardiovascular drug.

A minimum of twelve published clinical cohort studies have established a strong link between the use of statins and a lower incidence of epilepsy/seizures. These clinical cohort studies suggest that statins lower the likelihood of developing epilepsy not just after an ischemic stroke, but also after brain insults that occur as result of a variety of different causes such as a brain tumor or intracranial hemorrhage [4]. One of these studies examined data from the US Department of Veterans Affairs database for over a million people and discovered that those who used statins had a lower likelihood of epilepsy (OR = 0.64, 95% confidence interval [CI] = 0.56–0.73) [5]. There are eight distinct statins in clinical use and each has been studied for their possible anti-epileptogenic activity. Atorvastatin, however, was the most often used statin in the majority of clinical investigations, and it had the most statistically significant correlation with epilepsy risk reduction [6]. The question of whether seizures prevent or diminish the effects of statins after discontinuation remains unanswered; however, one retrospective clinical cohort study by Etminan M et al. attempted an analysis on this and found no benefits for past statin users, although it had only 14 cases in this category [7]. Several studies indicate that higher the statin use, the stronger the ant epileptogenic impact. According to one study, those who used statins for more than 2 weeks had a considerably reduced risk of post-stroke epilepsy than people who took statins for less than 2 weeks [8]. A meta-analysis conducted by Acton et al. demonstrated a statistical association between the usage of post-stroke statins in decreasing the incidence of early onset seizures and post-stroke epilepsy [9]. Furthermore, according to a study by Marcos et al. statins are associated with decreased mortality after status epilepticus [10]. Statin's anti-convulsive activity has been explained by suppressing reactive astrogliosis with neuroinflammation during crises; stimulating GABAergic activity while inhibiting glutamatergic; modulating the glycogen synthase kinase-3 pathway; and decreasing monocyte infiltration in hippocampus neuronal death and pro-inflammatory gene expression. These lipophilic statins may readily diffuse past the blood brain barrier and reach the central nervous system. Furthermore, monocarboxylic acid transporters in the blood brain barrier provide an alternate route for statins [11].

According to the recent clinical trials and studies, statins have been shown to be more potent and widely used because they are not only available globally but are also economically friendly. Statins are safe and well tolerated and they have been shown to be anti-epileptogenic in a broader variety of brain injuries than any other individual or class of substance; and they are the only compounds with both retrospective and prospective clinical evidence of potential anti-epileptogenic activity. To authorize one of these statins to be used clinically for antiepileptogenesis, its antiepileptogenic impact must be demonstrated in future time- and resource-intensive clinical studies. As a result, it is preferable to conduct a study of the existing research to decide which of the statins is the most reasonable alternative for antiepileptogenic therapy.

## Ethical approval

NA.

## Sources of funding

NA.

## Author contribution

All authors meet the inclusion criteria, and all authors read and approved the final version of the manuscript.

## Trail registry number

1. Name of the registry: NA.

2. Unique Identifying number or registration ID: NA.

3. Hyperlink to your specific registration (must be publicly accessible and will be checked): NA.

## Guarantor

Mohammad Mehedi Hasan.

Department of Biochemistry and Molecular Biology, Faculty of Life Science, Mawlana Bhashani Science and Technology University, Tangail, 1902, Bangladesh. Email: mehedi.bmb.mbstu@gmail.com.

## Annals of medicine and surgery

The following information is required for submission. Please note that failure to respond to these questions/statements will mean your submission will be returned. If you have nothing to declare in any of these categories then this should be stated.

## Consent

NA.

## Declaration of competing interest

NA.
